# The cost of scientific excellence – could it be expensive and out of reach?

**DOI:** 10.3325/cmj.2016.57.413

**Published:** 2016-10

**Authors:** Srećko Gajović, Roland Pochet

**Affiliations:** 1 University of Zagreb School of Medicine, Croatian Institute for Brain Research, Zagreb, Croatia *srecko.gajovic@hiim.hr*; 2 Université Libre de Bruxelles, Fac Medicine, Histologie, Brussels, Belgium

There is a considerable effort to understand the complex relation between the amount of financial investments and the corresponding research outcomes and achieved outputs. More and more, researchers are asked to prepare a detailed budget plan, and justify the used financial resources. The funding agencies, professional organizations, investors, industries, and governments on the other hand are under pressure to be savvy with their spending and they are keen to allocate the money in the most efficient way to reach their goals (1).

It is unanimously accepted that investing in science and research would generate the outputs which in turn would boost the economy, provide better health care, and increase the quality of life, ie, that investing in science correlates with important societal benefits. The problem is that the output and the time it takes to make “a product” out of the research project is not known but generally represents a long run effort with many trials and errors (2). Therefore, it becomes a political/economic issue linked to the vision that a country has about investing in science. There is increasing scrutiny over whether the influx of money will result in desired outcomes, and if the problems could be solved by an increase or decrease in the amount of financial resources.

Although scientific excellence is a frequently used term, it has a wide spectrum of meanings. It is frequently used as one of the judging criteria for issuing grants or awarding positions, nevertheless its definition remains elusive (3). Being part of job or grant applications, the section dealing with scientific excellence mainly considers the valorization of previous research outcomes by academic peers in the sense of novelty and impact.

When referring to scientific excellence, we often mix excellence with research outcomes, which are two different things. Indeed, to prove and document scientific excellence one would use research outcomes, eg, the list of publications, citations, patents, grants, collaborations, etc. These are the results of an individual, research group, institution, or country activities. Still, when speaking about scientific excellence, it could be better to consider the potential rather than realization. This difference is crucial to connect the financial investments in science with scientific excellence. If we could claim that scientific excellence is (at least partially) financially independent, then the emerging scientific communities with low levels of financial investments could still be perceived as a reservoir of scientific excellence. On the other hand, if it is (mainly) financially dependent, then low levels of investments in science and financial and economic crisis deplete the communities of scientific excellence and excellence starts to be a privilege of high-income geographical regions (4).

Such concept of scientific excellence, having a potential to solve important research questions, is of particular importance to the *Croatian Medical Journal*. Indeed, our editorial policy is directed to emerging scientific communities with an aim to serve as their door to the mainstream science. This means that we are keen to recognize the potential (ie, scientific excellence) in the contributions coming from these geographical regions and invest an extra effort to assist the authors to improve their manuscripts and make an impact at the global level. The prosperity of our journal depends on revealing the potential excellence and publishing it, in the hope that these valuable articles will be visible and cited, subsequently increasing the journal impact factor.

Still, if scientific excellence depends on financial spending, it could be that the journal is oriented in the wrong direction, searching for excellence in the places unable to cultivate it. Moreover, if scientific excellence is measured by the research realization and not by its potential, the indicators of realization would imply that there is a clear difference in scientific excellence among countries (4). Therefore, to disconnect scientific excellence from available financial resources, and assuming that it is (at least partially) financially independent, this article supports the argument that scientific excellence is a potential and that it cannot be measured by research outcomes (only). It can be hidden by unfavorable conditions, therefore it deserves the effort to find it, reveal it, and bring this potential to its fruitful realization.

Those performing their research in financially unfavorable conditions frequently claim that these conditions actually induce them to develop their excellence. They are driven by limited financial resources to carefully choose the study design and use innovativeness to reach their research goals despite the infrastructural obstacles. The results obtained include specific intellectual effort, which could qualify as an excellence similar to the one measured by scientific outputs.

The true nature of scientific excellence is probably somewhere between these two extremes of financial dependence vs independence, and the issue is worth investigating as it could be crucial for the scientific and educational strategies of both developed and developing countries. Recently the Aarhus declaration (5) called to more investments in scientific education of young talents, fearing that ongoing economic and financial crisis would undermine their development. This highlights that although different communities across the world can count on similar percentages of talents, whether these talents would turn to be well educated researchers achieving scientific excellence depends on whether the educational system is supported by financial resources. This is critical in PhD education, where students are expected to conduct their own research, which is frequently costly. Subsequently, even if we would assume that Spartan education, where the student is challenged by conditions without resources, could produce the best results, the criteria to obtain a PhD should not be compromised. Regrettably, the PhD criteria differ across the countries from a mere judgment by the local peers to the requirement to publish several articles in journals with high impact factors (counteracted by harmonization efforts, eg, Zagreb declaration) (6). There is an inevitable and important educational step from being a talent to being excellent in science. The selection of talents and their transformation to researchers with the potential of achieving excellence cannot be substituted or neglected, and this is the very weak spot of the low-investing scientific communities.

The world of financial inequalities and divisions frequently puts those who achieve scientific excellence in difficult position regardless of whether we connect excellence with the financial investment or not. If we claim it is financially independent, there is a risk of negligence due to the lack of resources. If we claim it is dependent, scientific excellence is in danger of rationalization of the use of resources. Those achieving scientific excellence can be perceived as draining the limited resources for something a community cannot afford. The connection between excellence and cost opens the question of what is the highest cost of excellence a community can pay for. Subsequently, a community with low financial resources can decide to cap the scientific excellence in order to rationally use the money.

In conclusion, the question of scientific excellence seems to be more significant for emerging than for developed research communities. The scarce financial resources need to be used efficiently and the potential of scientific excellence carefully measured, maintained, and increased. The *Croatian Medical Journal* is determined to ensure that the potential of excellence is transformed to open access, visible, and appreciated publications.
